# Prevalence and correlates of loneliness, perceived and objective social isolation during the COVID-19 pandemic. Evidence from a representative survey in Germany

**DOI:** 10.1007/s00127-022-02295-x

**Published:** 2022-04-27

**Authors:** André Hajek, Hans-Helmut König

**Affiliations:** Department of Health Economics and Health Services Research, University Medical Center Hamburg-Eppendorf, Hamburg Center for Health Economics, 20246 Hamburg, Germany

**Keywords:** Loneliness, Social isolation, Social exclusion, COVID-19, Corona, SARS-CoV-2

## Abstract

**Purpose:**

Our aim was to identify the prevalence and correlates of loneliness, perceived and objective social isolation in the German population during the COVID-19 pandemic.

**Methods:**

Data were taken from a representative survey with *n* = 3075 individuals (18–70 years; August/September 2021). Valid measures were used to quantify the outcomes (loneliness: De Jong Gierveld scale; perceived social isolation: Bude/Lantermann tool; objective social isolation: Lubben Social Network Scale). Multiple logistic regressions were used to identify the correlates of these three outcomes.

**Results:**

The prevalence of loneliness was 83.4%, the prevalence of perceived social isolation was 59.1% and the prevalence of objective social isolation was 28.9%. The prevalence rate significantly differed between the subgroups (e.g., the prevalence of perceived social isolation was 73.9% among individuals aged 18–29 years, whereas it was 48.8% among individuals aged 60–70 years). In regression analysis, several correlates of these outcomes were identified (e.g., marital status, age group (with changing signs), migration background, sports activities, or self-rated health).

**Conclusion:**

Our study particularly identified very to extraordinarily high prevalence rates for social isolation and loneliness, respectively. Knowledge about the correlates (e.g., age group) may help to address these individuals during the ongoing pandemic.

**Supplementary Information:**

The online version contains supplementary material available at 10.1007/s00127-022-02295-x.

## Introduction

Loneliness refers to a perceived discrepancy between actual and desired social contacts [1]. Perceived social isolation refers to a feeling of not belonging to the society [2]. Moreover, objective social isolation refers to a limited number of social contacts (e.g., clubs) [3]. All of these factors can be considered as unfulfilled basic social needs [4]—mainly referring to the need for emotional and social connectedness [4]. Different social need theories have been presented such as loneliness theories [5], self-determination theory [6] and the theory of Social Production Functions—please see Bunt et al. for a detailed overview [4].

Such unfulfilled social needs can contribute to morbidity and mortality [7, 8]. Thus, it is of importance to identify the prevalence and the correlates of these social needs. Loneliness, perceived social isolation and objective social isolation are interrelated, but do not measure the same (please also see the results section) [9]. For example, individuals can feel lonely without feeling socially isolated (and vice versa). Moreover, they differ in their antecedents [10] and consequences [11].

For example, in a cross-sectional study (with data collection from August 2011 to November 2014), a prevalence rate of 12.3% (95% CI 11.6–13.0) was reported for objective social isolation in the city Leipzig (Germany) [12] using the Lubben Social Network Scale [13] (6 item version; score below 12 was considered as an indicator of objective social isolation).

Based on some objective indicators, somewhat lower isolation scores were reported among the adult population in Germany in the year 2011 [14]. A study using data from Australian adults (year 2004; *n* = 3015 randomly sampled participants of the South Australian Health Omnibus Survey; response rate of 72%) showed that about 9% reported some social isolation and 7% reported feeling isolated or very isolated (based on the Friendship Scale) [15]. Based on data from the Swiss Health Survey (year 2012; *n* = 21,597 randomly sampled individuals; response rate of 53%; sample which was weighted and calibrated to ensure representativeness and take into consideration the proportion of non-responders) 7.7% of the Swiss population aged 15 years and older are socially isolated (according to an integration index) [16].

Particularly in times of the pandemic (with its specific conditions such as social distancing), it appears to be plausible that the prevalence of loneliness, perceived social isolation and objective social isolation is markedly higher compared to the times prior to the pandemic. Recent studies have actually shown that loneliness particularly increased during the first partial lockdown (March to May 2020) in Germany [17, 18]. However, far less is known about the prevalence and correlates 1.5 years later (i.e., late Summer 2021). Thus, the aim of this present study was to identify the prevalence and correlates of loneliness, perceived and objective social isolation in the German population in late Summer 2021. It may be the case that individuals adapted to the conditions of the pandemic (‘habituation’ [19]), and thus the prevalence rate of loneliness, perceived and objective social isolation might be comparable or only somewhat higher compared to prior to the pandemic. However, as initially stated, we assume that most of the individuals struggle to adapt to these conditions over time and might rather report high prevalence rates for loneliness, perceived and objective social isolation (compared to the time prior and also compared to the time at the beginning of the pandemic). However, it is worth noting that we did not expect any specific prevalence rates.

Thus far, there is limited knowledge regarding the prevalence and correlates of loneliness, perceived and objective social isolation from late Summer 2021 based on data covering the general adult population in Germany. Moreover, it should be stressed that previous German studies commonly did not report the prevalence rates and did not simultaneously quantify loneliness, perceived as well as objective social isolation. Knowledge about the prevalence can assist in clarifying whether these social needs reflect a challenge during the COVID-19 pandemic. Moreover, identifying the correlates of these social needs may help to address individuals at risk for loneliness, perceived and objective social isolation during the pandemic.

A brief description of the COVID-19 pandemic (and particularly the measures) in Germany is provided to help readers better understand the situation: National efforts to halt the spread of COVID-19 began in mid-March 2020 (e.g., closing of schools). Some restrictions were relaxed in mid-April 2020. Schools reopened in May of 2020. In the months that followed, additional restrictions were relaxed. Since a significant increase in infection rates was observed in autumn 2020, several restrictions have been imposed. The restrictions were relaxed in May 2021 and remained quite stable until late Summer 2021 (time of data collection for this present study). More precisely, during the time of data collection, it was mandatory to be vaccinated, recovered, or tested for anyone who meets in publicly accessible indoor spaces in Germany. Visits to restaurants, cinemas, hairdressers and other body-related services, gyms, swimming pools and sports halls, events, hospital, rehabilitation or disabled facilities, and nursing homes were all subject to testing. A negative rapid test within the last 24 h or a PCR test was required. The results of PCR tests were valid for 48 h. Those staying in hotels also had to show a negative test. It had to be done every third day of a stay.

## Materials and methods

### Sample

Data were taken from a representative online survey (*n* = 3075 adults aged 18–70 years; living in Germany). Only younger (≤ 17 years) or older individuals (≥ 71 years) and individuals not living in Germany were excluded from participation. It should be noted that the questionnaire was exclusively available in German language. Fieldwork was carried out from late August to early September 2021. A well-established market research company (respondi) recruited the individuals (using its own online access panel). Members of this panel are recruited via campaigns conducted by respondi itself. For groups that are difficult to reach (e.g., individuals in old age, ethnic minorities) multiple recruitment sources were used by respondi such as online campaigns, cooperation agreements or search engine marketing. Based on this sampling frame, individuals were drawn in such a way reflecting the distribution of gender, age bracket and federal state in the German adult population [20]. Using the socio-demographic data, a random sample of the online access panel was drawn. Approximately 14,000 individuals received an invitation to participate in this survey (response rate: 22%). After the first invitation, individuals were reminded up to two times (time span between the reminders were 2 or more days). Digital fingerprint solutions were used to avoid duplicates. Due to data availability, it was not possible to compare participants and non-participants (e.g., regarding age group, income, education or health-related factors).

Informed consent was provided. The study was approved by the Local Psychological Ethics Committee of the Center for Psychosocial Medicine of the University Medical Center Hamburg-Eppendorf (number: LPEK-0356).

### Outcomes

The De Jong Gierveld Loneliness scale was used to assess loneliness (6-item version; three items were recoded) [21]. The items were averaged to create a score (0 to 6; higher values reflect higher loneliness). As recently suggested by van Tilburg and De Jong Gierveld [22], scores of 0–1 were used to classify individuals as ‘not lonely’ and higher scores were used to classify individuals as ‘lonely’. In our study, Cronbach’s alpha was 0.78. The scale has favorable psychometric properties [21, 23].

To quantify perceived social isolation, the tool developed by Bude and Lantermann [2] (4-items) was used. By averaging the items, a score was calculated (0–6, higher values reflect higher perceived social isolation). Likewise, scores of 0–1 were used to classify individuals as ‘not socially isolated (perceived)’ and scores above were used to classify individuals as ‘socially isolated (perceived)’. Cronbach’s alpha was 0.92 in our study.

The Lubben Social Network Scale (6-item version) [13] was used to measure objective social isolation. The sum score ranges from 0 to 30 (higher values corresponding to lower objective social isolation). As recommended, a score below 12 was used to classify individuals as ‘socially isolated (objective)’, whereas higher values were used to classify individuals as ‘not socially isolated (objective)’ [13]. Cronbach’s alpha was 0.87 in our study. Good psychometric properties have been reported [13].

### Independent variables

As correlates, we included the following factors in logistic regressions: Sex (women; men; diverse), age group (18–29 years; 30–39 years; 40–49 years; 50–59 years; 60 years and older), presence of at least one child in own household (no; yes), marital status (married, living together with spouse; married, not living together with spouse; divorced; widowed; single), education (upper secondary school; qualification for applied upper secondary school; polytechnic Secondary School; intermediate Secondary School; Lower Secondary School; currently in school training/education; without school-leaving qualification), and occupational status (full-time employed; retired; other), migration background (no; yes), and labor force participation (full-time employed; retired; other). Additionally, lifestyle-related correlates were included in our regression model: alcohol intake (daily; several times per week; once a week; 1–3 times per month; less often; never), smoking status (yes, daily; yes, sometimes; no, not anymore; never smoker), and sports activities (no sports activity; less than 1 h a week; regularly, 1–2 h a week; regularly, 2–4 h a week; regularly, more than 4 h a week). Moreover, health-related correlates were included in the regression model: presence of one or more chronic conditions (no; yes), self-rated health (single item measure ranging from 1 = very bad to 5 = very good) and vaccination against COVID-19 (no; yes).

### Statistical analysis

First, prevalence of loneliness, perceived social isolation and objective social isolation was shown for some groups (i.e., stratified by sex, age brackets, education, having children, migration background, vaccination against COVID-19, and chronic diseases). Subsequently, an overview about the combination of the different measures (loneliness, perceived social isolation and objective social isolation) is given. Thereafter, multiple logistic regressions were used to identify the correlates of loneliness, perceived social isolation and objective social isolation.

No missing data were present in our observed variables. Thus, imputation techniques were not used. Moreover, weights were not used because the sample matches our target cohort in terms of state, age group and gender (please see Supplementary Table 1).

Statistical significance was defined as p value of 0.05 or smaller. Stata 16.1 (Stata Corp., College Station, Texas) was used to conduct statistical analyses.

## Results

### Sample characteristics

The total sample consisted of 1570 (51.1%) female individuals and the average age was 44.5 years (SD: 14.8 years). The prevalence for loneliness, perceived social isolation and objective social isolation is shown in Table 1 (in total and stratified by subgroups). The average loneliness score was 2.5 (SD: 1.3), the average perceived social isolation score was 1.8 (SD: 1.6) and the average objective social isolation score was 14.7 (SD: 6.1). Please also see the box plots (Supplementary Files 2–4).

**Table 1:** Prevalence of loneliness, perceived isolation and objective social isolation in different groups

	*n*	Loneliness (%)	*p* value	Perceived social isolation (%)	*p* value	Objective social isolation (%)	*p* value
Total sample	3075	83.4		59.1		28.9	
Gender			0.26		< 0.01		< 0.01
Male	1502	84.4		55.8		31.4	
Female	1570	82.4		62.1		26.7	
Diverse	3	100.0		100.0		0.0	
Age group			0.06		< 0.001		< 0.001
18–29 years	628	86.5		73.9		18.0	
30–39 years	597	83.1		62.3		24.8	
40–49 years	597	84.8		56.6		31.5	
50–59 years	659	81.2		53.4		34.4	
60 years and older	594	81.5		48.8		36.0	
Children in own household			0.70		0.41		< 0.001
No	2206	83.5		59.5		32.0	
Yes	869	83.0		57.9		21.3	
Marital status			< 0.001		< 0.001		< 0.001
Single		90.4		69.4		34.3	
Divorced		90.8		61.9		42.7	
Widowed		87.5		47.5		35.0	
Married, not living together with spouse	1313	84.6		67.7		20.8	
Married, living together with spouse	1762	78.7		53.5		24.8	
Education			0.46		0.46		< 0.001
Upper secondary school	1326	82.7		58.4		21.9	
Qualification for applied upper secondary school	328	83.5		58.2		25.0	
Polytechnic Secondary School	168	79.8		55.4		35.1	
Intermediate Secondary School	888	84.1		59.9		33.8	
Lower Secondary School	347	85.6		61.1		44.4	
Currently in school training/education	9	100.0		88.9		0.0	
Without school-leaving qualification	9	77.8		66.7		55.6	
Migration background			< 0.001		< 0.001		0.67
No	2724	82.5		57.6		28.8	
Yes	351	90.0		70.4		29.9	
Employment status			0.03		< 0.001		< 0.001
Full-time employed	1458	82.0		53.8		23.9	
Retired	499	82.2		54.3		42.3	
Other	1118	85.7		68.1		29.5	
Vaccinated against COVID-19			0.06		< 0.01		0.01
No	593	86.0		63.9		33.2	
Yes	2482	82.8		57.9		27.9	
Chronic diseases			0.08		< 0.01		< 0.001
Absence of at least one chronic disease	1765	82.4		56.5		24.0	
Presence of at least one chronic disease	1310	84.7		62.5		35.6	

Chi^2^ tests were conducted (p-values). Individuals were classified as ‘lonely’ when their score was greater than 1. Analogously, individuals were classified as ‘socially isolated (perceived)’ when their score was greater than 1. Additionally, individuals were classified as ‘socially isolated (objective)’ when their score was below 12

The prevalence of loneliness was 83.4%, the prevalence of perceived social isolation was 59.1% and the prevalence of objective social isolation was 28.9%. The prevalence rates markedly differed between subgroups. For example, the prevalence of loneliness was 90.0% among individuals with a migration background, whereas it was 82.5% among individuals without a migration background. A second example: the prevalence of perceived social isolation was 73.9% among individuals aged 18–29 years, whereas it was 48.8% among individuals aged 60–70 years. In contrast, the prevalence of objective social isolation was 18.0% among individuals aged 18–29 years, whereas it was 36.0% among individuals aged 60–70 years.

With regard to the prevalence of loneliness, significant differences in the subgroups existed according to marital status, migration background, and employment status. Moreover, the prevalence of perceived social isolation significantly differed according to gender, age group, marital status, migration background, employment status, vaccination against COVID-19 and chronic diseases. Additionally, the prevalence of objective social isolation significantly differed according to all groups (except for migration background). Further details are given in Table 1.

Table 2 gives an overview about the combination of the different measures (loneliness, perceived social isolation and objective social isolation). In sum, 20.5% of the individuals reported being lonely and being socially isolated (both, perceived and objective). Furthermore, 20.7% of the individuals reported being lonely, but were not socially isolated (both perceived and objective). Additionally, 2.7% of the individuals reported being socially isolated (perceived), but were not lonely and socially isolated (objective). Furthermore, 1.3% of the individuals were socially isolated (objective), but were not lonely and socially isolated (perceived). Moreover, 12.2% of the individuals did not feel lonely and were not socially isolated (both, perceived and objective). Further details are shown in Table 2.

**Table 2:** Loneliness, perceived isolation and objective social isolation

	Absence of objective social isolation		Presence of objective social isolation	
	Absence of perceived social isolation	Perceived social isolation	Absence of perceived social isolation	Presence of perceived social isolation
Absence of loneliness	375	84	39	13
Presence of loneliness	638	1088	207	631

Absolute frequencies are given to ensure readability. How to read this table: For example, among individuals with objective social isolation: 39 individuals were not lonely and not socially isolated (perceived), 13 individuals were not lonely and socially isolated (perceived), 207 individuals were lonely and not socially isolated (perceived) and 631 individuals were lonely and socially isolated (perceived)

In our current study, the association between loneliness (measured continuously) and perceived social isolation (measured continuously) was *r* = 0.56 and the association between loneliness and objective social isolation (measured continuously) was *r* = − 0.44 (worth repeating because higher scores on the Lubben Social Network Scale refer to lower objective social isolation). Furthermore, the association between perceived social isolation and objective social isolation was *r* = − 0.27.

The associations between the dichotomized outcomes were as follows: Cramer’s *V* = 0.37 (association between loneliness and perceived social isolation), Cramer’s *V* = 0.18 (association between loneliness and objective social isolation), and Cramer’s *V* = 0.17 (association between perceived social isolation and objective social isolation).

### Regression analysis

Results of multiple logistic regressions are depicted in Table 3. A higher likelihood of loneliness was associated with being male, younger age group, being single, being divorced or being widowed (compared to ‘being married, living together with spouse’), having a migration background, performing no sports activity (compared to more than 4 h a week of regular sports activities) and low self-rated health. Similarly, a higher likelihood of perceived social isolation was associated with younger age group, being single or being divorced (compared to ‘being married, living together with spouse’), having a migration background, not being full-time employed or retired, never smoking (compared to smoking daily), never drinking alcohol (compared to drinking less often than 1–3 times a month) and low self-rated health. A higher likelihood of objective social isolation was associated with higher age group, not having children in own household, being single or being divorced (compared to ‘being married, living together with spouse’), having a lower educational level, not being full-time employed (i.e., retired or other), performing no sports activity, never drinking alcohol, and low self-rated health.

**Table 3:** Correlates of loneliness, perceived social isolation and objective social isolation. Results of multiple logistic regressions

	(1)	(2)	(3)
Independent variables	Loneliness	Perceived social isolation	Objective social isolation
Sex—Women (Ref.: Men)	0.74** (0.59–0.92)	0.93 (0.78–1.11)	0.86 (0.71–1.04)
Diverse	–	–	–
Age group: 30–39 years (Ref.: 18–29 years)	0.78 (0.56–1.11)	0.63*** (0.48–0.82)	1.78*** (1.31–2.43)
40–49 years	0.83 (0.57–1.19)	0.44*** (0.33–0.59)	2.49*** (1.81–3.42)
50–59 years	0.58** (0.41–0.83)	0.32*** (0.24–0.43)	2.18*** (1.58–3.00)
60 years and older	0.61* (0.39–0.94)	0.25*** (0.18–0.35)	1.79** (1.23–2.61)
Children in own household—Yes (Ref.: No)	1.18 (0.92–1.50)	1.03 (0.85–1.25)	0.61*** (0.49–0.75)
Marital status—Single (Ref.: married, living together with spouse single/divorced/widowed/)	2.35*** (1.78–3.12)	1.44*** (1.18–1.77)	1.54*** (1.24–1.90)
Divorced	2.71*** (1.69–4.34)	1.46* (1.08–1.97)	1.49** (1.10–2.02)
Widowed	2.26* (1.12–4.56)	0.94 (0.58–1.53)	1.12 (0.67–1.86)
Married, not living together with spouse	1.40 (0.84–2.32)	1.44 + (0.96–2.16)	0.85 (0.53–1.35)
Migration—Migration background (Ref.: no migration background)	1.83** (1.25–2.67)	1.43** (1.10–1.86)	1.28+ (0.98–1.67)
Highest educational degree—qualification for applied upper secondary school (Ref.: upper secondary school)	1.04 (0.74–1.46)	1.01 (0.78–1.32)	1.02 (0.75–1.37)
Polytechnic Secondary School	0.75 (0.49–1.17)	1.11 (0.77–1.58)	1.27 (0.88–1.85)
Intermediate Secondary School	1.07 (0.83–1.37)	1.12 (0.92–1.37)	1.34** (1.08–1.65)
Lower Secondary School	1.09 (0.75–1.58)	1.19 (0.91–1.58)	1.63*** (1.23–2.16)
Currently in school training/education	–	6.83+ (0.72–64.64)	–
Without school-leaving qualification	0.30 (0.06–1.53)	0.62 (0.15–2.63)	2.87 (0.65–12.61)
Employment status—Retired (Ref.: Full-time employed)	0.82 (0.57–1.18)	1.13 (0.85–1.50)	1.40* (1.05–1.87)
Other	1.11 (0.87–1.42)	1.38*** (1.15–1.67)	1.26* (1.03–1.55)
Smoking—Yes, daily (Ref: Never smoker)	0.89 (0.68–1.17)	0.79* (0.64–0.98)	0.95 (0.75–1.19)
Yes, sometimes	1.18 (0.77–1.79)	0.94 (0.70–1.27)	1.06 (0.76–1.47)
No, not anymore	0.97 (0.75–1.24)	0.82+ (0.67–1.00)	0.96 (0.77–1.19)
Sports activities—Less than one hour a week (Ref.: no sports activity)	0.90 (0.66–1.23)	1.10 (0.87–1.39)	0.80 + (0.64–1.02)
Regularly, 1–2 h a week	0.92 (0.68–1.25)	0.89 (0.71–1.11)	0.55*** (0.43–0.70)
Regularly, 2–4 h a week	0.92 (0.66–1.28)	0.86 (0.67–1.11)	0.59*** (0.44–0.78)
Regularly, more than 4 h a week	0.67* (0.48–0.94)	0.79+ (0.60–1.03)	0.66** (0.49–0.88)
Alcohol intake—Daily (Ref.: Never)	1.35 (0.81–2.25)	1.00 (0.69–1.44)	0.53** (0.36–0.78)
Several times a week	1.01 (0.72–1.43)	0.86 (0.66–1.12)	0.52*** (0.39–0.68)
Once a week	1.11 (0.78–1.58)	1.03 (0.79–1.35)	0.54*** (0.40–0.72)
1–3 times a month	0.78 (0.56–1.09)	0.83 (0.63–1.08)	0.55*** (0.41–0.73)
Less often	0.95 (0.70–1.31)	0.76* (0.60–0.97)	0.79+ (0.61–1.00)
Vaccinated against COVID-19: Yes (Ref.: No)	0.86 (0.65–1.12)	0.86 (0.70–1.06)	0.81+ (0.66–1.01)
Chronic diseases: Presence of at least one chronic disease (Ref.: Absence of chronic diseases)	0.87 (0.70–1.10)	1.05 (0.88–1.26)	0.96 (0.79–1.17)
Self-rated health (1 = very bad to 5 = very good)	0.53*** (0.46–0.62)	0.52*** (0.46–0.58)	0.70*** (0.63–0.78)
Constant	79.25*** (35.18–178.54)	37.02*** (19.95–68.72)	1.47 (0.79–2.74)
Observations	3063	3072	3063
Pseudo R^2^	0.077	0.097	0.103

Odds ratios are displayed. 95% CI in parentheses. ***p < 0.001, **p < 0.01, *p < 0.05, ^+^*p* < 0.10; ‘Diverse’ sex and ‘currently in school training/education’ (except for the model with perceived social isolation as outcome measure) were dropped as both of them predict the outcomes perfectly. Individuals were classified as ‘lonely’ when their score was greater than 1. Analogously, individuals were classified as ‘socially isolated (perceived)’ when their score was greater than 1. Additionally, individuals were classified as ‘socially isolated (objective)’ when their score was below 12

## Discussion

Our study extends previous knowledge by identifying the prevalence and correlates of loneliness, perceived and objective social isolation in the German population in late Summer 2021 (1.5 years after the first partial lockdown in Germany due to the COVID-19 pandemic). The prevalence of loneliness was 83.4%, the prevalence of perceived social isolation was 59.1% and the prevalence of objective social isolation was 28.9%. Several correlates of these outcomes were identified (e.g., marital status, age group (with changing signs), migration background, sports activities, or self-rated health).

With regard to the prevalence rates, studies prior to the pandemic showed markedly lower prevalence rates in Germany (e.g., prevalence of 12.3% for objective social isolation based on the Lubben Social Network Scale (6-item version) in Leipzig, Germany almost 10 years ago [12]). While some German studies also exist during the pandemic [17, 18, 24], these studies mostly showed an increase in loneliness scores during the first partial lockdown from March to May 2020 [17, 18] (as an overview: [25])—and did not focus on displaying the prevalence. For example, Bücker et al. [17] performed a daily diary study of 4844 participants in Germany between Mid-March and Mid-April 2020. Loneliness was quantified using four items ("I felt lonely today", "I felt left out today", "I had no one to turn to today", and "I felt isolated from the others today"). They recruited individuals by means of several online sources (such as Xing, Facebook and Twitter), media reporting and personal contacts; they acknowledged the fact that their study was not nationally representative. A key finding was that daily loneliness increased in the first 2 weeks, but decreased subsequently. Another German study (longitudinal experience sampling study) collected data from Mid-March to Mid-May 2020 (*n* = 529 participants, mainly students at the FernUniversität in Hagen (a distance learning university) [18]. They used the 11-item De Jong Gierveld loneliness scale to quantify loneliness. They showed that while physical loneliness was higher during the contact restrictions, emotional and social loneliness remained almost constant [18]. A study from Amsterdam (Amsterdamse Gezondheidsmonitor) also showed that loneliness (moderate and severe) only slightly increased from the year 2016 (48%) to September 2020 (53%) [26].

Our study adds to this knowledge by identifying the prevalence of loneliness, perceived and objective social isolation in the German adult population about 1.5 years later in the pandemic (August/September 2021). In general, it should be acknowledged that it is somewhat difficult to compare our present results to previous studies (both, during and before the pandemic) since several tools exist to quantify social isolation (such as the Integration index or the friendship scale) [27–29].

In our view, the quite unexpected very to extraordinarily high prevalence rates may be explained by the ongoing and apparently lasting influence of the pandemic and its conditions on the lives of the German adult population. For example, when comparing younger and older individuals, individuals 18–29 years reported high prevalence rates of loneliness and perceived social isolation, whereas individuals aged 60–70 years reported a high prevalence rate of objective social isolation. This may reflect that older individuals practice social distancing during the pandemic (and frequently did not replace these contacts by, e.g., technical solutions such as Skype). In contrast, younger individuals may more often stay in contact with friends and relatives (e.g., via technical solutions). However, these younger individuals particularly suffer from the pandemic. This supports very recent research showing particularly high prevalence rates of both depression and anxiety among younger individuals in Germany during the course of the pandemic [30, 31]. Due to financial hardship in young individuals, those individuals may restrict social activities and may thus feel excluded from the society [30]. Given the alarming prevalence rates identified in our study and the current lack of comparable studies, future in depth studies (e.g., based on qualitative data) are needed to better understand why individuals report such high loneliness and social isolation levels. Moreover, studies from other countries are important to be able to better classify our results internationally.

Our findings also confirm previous research showing that mental wellbeing remained largely unaltered during the first COVID-19 lockdown among older individuals (65+ years) in Germany [32]—which may be explained by resilience in this age bracket [32]. Such explanations given above may also explain the differences found in our regressions between the age groups. Previous research conducted in Canada during the pandemic also showed higher loneliness scores in younger adults compared to individuals aged 60 years and above [33]. Similar results were also identified in early 2021 in the German population [34].

Our study showed that being married and living together with spouse is associated with a lower likelihood of loneliness, perceived and objective social isolation. This is well in line with previous research prior [35] and during the pandemic [36]. This appears to be very plausible given the fact that such a relationship can easily assist in maintaining a personal, social contact during times of social distancing. It seems that such a relationship can contribute to avoid loneliness, perceived and objective social isolation.

Having a migration background was associated with a higher likelihood of loneliness and perceived isolation in our study, whereas the association with objective isolation was only marginally significant (*p* = 0.07). A recent German study conducted from January to February 2021 also showed higher loneliness scores among individuals with a migration background [34]. The association with loneliness and perceived social isolation may be explained by the fact that individuals with a migration background may neither be able to travel to family and friends living abroad nor to receive such visits—both due to travel restrictions [37]. However, previous research already demonstrated that individuals with a migration background reported higher loneliness scores despite having comparable number of social contacts prior to the pandemic (in comparison to individuals without a migration background [38]). Thus, beyond these travel restrictions, other factors may be important here. We assume that there may be differences in the quality of the relationships between individuals with a migration background and their counterparts. For example, individuals with a migration background may miss local networks or close relatives living abroad [39]. These factors may increase feelings of loneliness and not belonging to the society.

We found that sports activities were associated with a lower likelihood of loneliness and objective social isolation, whereas the association with perceived social isolation was only marginally significant (*p* = 0.09). Such sports activities may first help to alleviate feelings of loneliness during the pandemic (e.g., via team sports activities) [40]. Moreover, being involved in sports activity may help to avoid objective social isolation via maintaining social contacts (e.g., even in times of social distancing through digital sports lessons) [40].

The association between lower self-rated health and a higher likelihood of loneliness, perceived and objective social isolation which was found in our study is in accordance with studies both prior [41] and during the pandemic [42]. It appears very plausible and is often explained by the fact that a low self-rated health is associated with reduced social activities and social withdrawal [41].

Interestingly, not being vaccinated against COVID-19 is (marginally significantly: *p* = 0.06) associated with a higher likelihood of objective social isolation. Such individuals may feel stigmatized from the society [43]. Moreover, friends or relatives may at least temporarily distance themselves from such individuals because of the transmission risk.

Quite surprisingly, more frequent alcohol intake was associated with a lower likelihood of objective social isolation in our study. It may be the case that drinking alcohol sometimes reflect social activities during the pandemic. However, this association should be treated with great caution since the reference category ‘never’ (self-reported) cannot distinguish between ex-drinkers and non-drinkers [44]. Additionally, the frequency of alcohol intake may not necessarily correspond to the volume of alcohol consumption [44].

A few strengths and limitations are worth noting. Data were taken from a large, representative sample. However, it should be acknowledged that the questionnaire was only available in German language which excluded individuals with insufficient German language skills. Furthermore, we cannot dismiss the possibility of a sample selection bias. While our online sample matches the German population, e.g., in terms of age group, sex, state and factors such as median income and proportion of unemployed individuals, the proportion of individuals with a university degree was a little higher in our sample (25.4%) compared to the general adult population in Germany (18.5%) [45]. Thus, higher educated individuals may be overrepresented in our current sample which should be noted when generalizing our findings. Moreover, previous research has shown that online panels can be selective in terms of including individuals with spare time, but socially engaged [46, 47].

Valid tools were used to assess the outcomes. Since this is a cross-sectional study, it is difficult to draw causal conclusions. Moreover, our study focused on individuals aged 18–70 years. More research is required focusing on other groups (e.g., adolescence or oldest old individuals).

In conclusion, our study particularly identified very to extraordinarily high prevalence rates for social isolation and loneliness, respectively. Knowledge about the correlates (e.g., age group) may help to address these individuals during the ongoing pandemic.

## Supplementary Information

Below is the link to the electronic supplementary material.

Supplementary file1 (DOCX 30 KB). Comparison of the target quote and our sample

Supplementary file2 (PDF 45 KB). Loneliness (box plot)

Supplementary file3 (PDF 45 KB). Perceived social isolation (box plot)

Supplementary file4 (PDF 45 KB). Objective social isolation (box plot)

## Data Availability

The datasets used and analysed during the current study are available from the corresponding author on reasonable request for all interested researchers.
