# Supplementary figures and images for: Prevalence and correlates of loneliness, perceived and objective social isolation during the COVID-19 pandemic. Evidence from a representative survey in Germany
Source: Soc Psychiatry Psychiatr Epidemiol. 2022 Apr 27;57(10):1969–78. doi: 10.1007/s00127-022-02295-x (PMC9043881; doi:10.1007/s00127-022-02295-x)

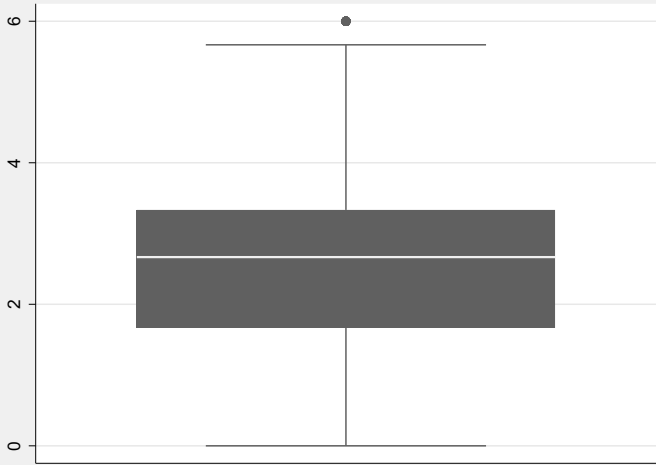

Supplement: Supplementary file 2 — Supplementary file2 (PDF 45 KB). Loneliness (box plot) [file 127_2022_2295_MOESM2_ESM.pdf]

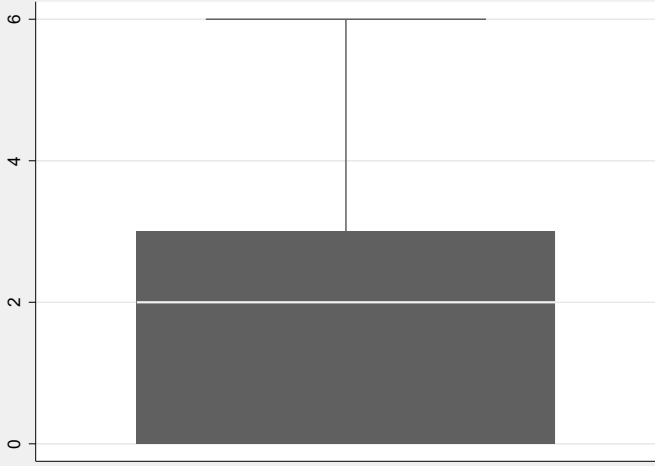

Supplement: Supplementary file 3 — Supplementary file3 (PDF 45 KB). Perceived social isolation (box plot) [file 127_2022_2295_MOESM3_ESM.pdf]

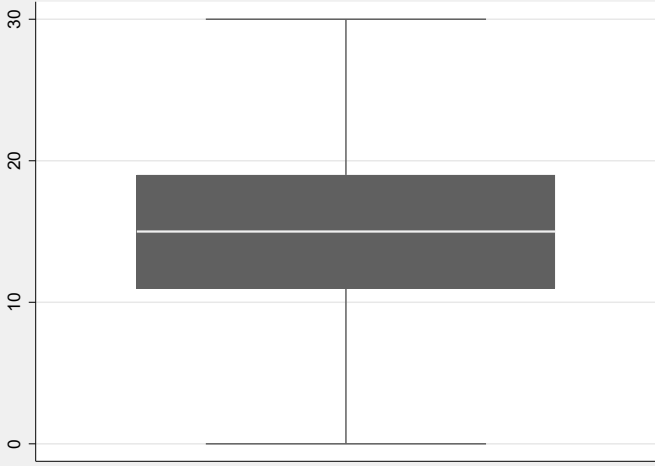

Supplement: Supplementary file 4 — Supplementary file4 (PDF 45 KB). Objective social isolation (box plot) [file 127_2022_2295_MOESM4_ESM.pdf]
